# A novel class I HDAC inhibitor, MPT0G030, induces cell apoptosis and differentiation in human colorectal cancer cells via HDAC1/PKCδ and E-cadherin

**DOI:** 10.18632/oncotarget.2155

**Published:** 2014-07-01

**Authors:** Li-Ting Wang, Jing-Ping Liou, Yu-Hsuan Li, Yi-Min Liu, Shiow-Lin Pan, Che-Ming Teng

**Affiliations:** ^1^ Pharmacological Institute, College of Medicine, National Taiwan University, Taipei, Taiwan; ^2^ School of Pharmacy, College of Pharmacy, Taipei Medical University, Taipei, Taiwan; ^3^ The Ph.D. Program for Cancer Biology and Drug Discovery, College of Medical Science and Technology, Taipei Medical University, Taipei, Taiwan

**Keywords:** MPT0G030, PKCδ, E-cadherin, HDAC, differentiation

## Abstract

Accumulation of genetic and epigenetic changes contributes to cancer development and progression. Compared with gene mutations or deletions, epigenetic changes are reversible, which alter the chromatin structure remodeling instead of changes in DNA sequence, and therefore become a promising strategy for chemotherapy. Histone deacetylases (HDACs) are a class of enzymes that responsible for the epigenetic regulation of gene expression. MPT0G030 is a potent and selective class I HDAC inhibitor which showed broad-spectrum cytotoxicity against various human cancer cell lines. *in vitro* fluorometric HDAC activity assay showed that MPT0G030 effectively inhibited Class I HDACs (HDAC1~3), which were overexpressed in many malignant neoplasms. Interestingly, MPT0G030 not only induced histone acetylation and tumor suppressor p21 transcription, but also redistributed E-cadherin and activated Protein Kinase C δ (PKCδ), which was linked to cell apoptosis and differentiation. Further, activation of PKCδ was demonstrated to be modulated through HDAC1. The *in vivo* anticancer activity of MPT0G030 and the importance of PKCδ were confirmed in the HT-29 tumor xenograft models. Taken together, those results indicate that MPT0G030, a class I HDAC inhibitor, has great potential as a new drug candidate for cancer therapy.

## INTRODUCTION

Colorectal cancer is the third most common malignant neoplasm worldwide. In contrast to other cancers, the colorectal adenoma-carcinoma sequence is well established as an ordered process of sequential accumulation of genetic and epigenetic changes in tumor suppressor genes and oncogenes [1]. Compared with gene mutations or deletions, epigenetic changes, which are alterations of chromatin structure rather than changes in DNA sequence, are reversible. Therefore, they can be used as a promising strategy for chemotherapy. Histone deacetylases (HDACs) comprise a class of enzymes responsible for the epigenetic regulation of gene expression, and play critical roles in cellular processes including cell proliferation, apoptosis, differentiation and angiogenesis [2]. Numerous reports indicate HDACs are overexpressed in many cancers, especially HDAC1, HDAC2, and HDAC8 are overexpressed in colon cancer, and inhibit specific tumor suppressor genes, resulting in an aberrant epigenetic status compared to adjacent normal cells [3, 4]. Therefore, HDAC inhibitors have the potential to become a new class of chemotherapy drugs for cancer treatment.

Interestingly, HDAC families play critical roles in regulating normal homeostasis of colonic epithelium cells, especially in the proliferative crypt compartment. Considerable evidence shows that that knockdown of class I HDACs 1 and 2 inhibits cell growth and survival in various colon cancer cell lines [5-7]. In contrast to cytotoxic chemotherapeutic drug treatment, HDAC inhibitor treatment of colon cancer cells results in enhanced differentiation and accelerated apoptosis [4, 8]. Thus, differentiation therapy has been recently proposed as an alternative medical treatment for colon cancer chemotherapy.

Protein Kinase C (PKC) family consists of numbers of serine-threonine protein kinases involved in regulating diversified signaling transduction cascades. Activation of PKCα and β isoforms contributes to tumor proliferation, invasion, drug resistance, and genetic instability [9]. However, many studies have implicated downregulation, rather than activation, of PKCδ in sporadic human colonic cancer in resistance to apoptosis and differentiation [9, 10]. Transient overexpression of PKCδ has been linked to tumor-suppressive functions, including suppression of anchorage-independent cell growth [11], reversal of colonic epithelial cells transformation via Src [12], and inhibition of neoplastic phenotype via p53 [13]. Therefore, PKCδ is thought of as a tumor suppressor. Moreover, accumulating evidence suggests that HDAC inhibitors could activate protein transcription through protein kinase signaling pathways, rather than chromatin remodeling. It has been shown HDAC inhibitor modulates Sp1-dependent gene expression through the PKCδ signaling pathway [14]. However, the degree and mechanism underlying the HDAC involvement in protein kinase activation remain unexplored.

**Figure 1:**
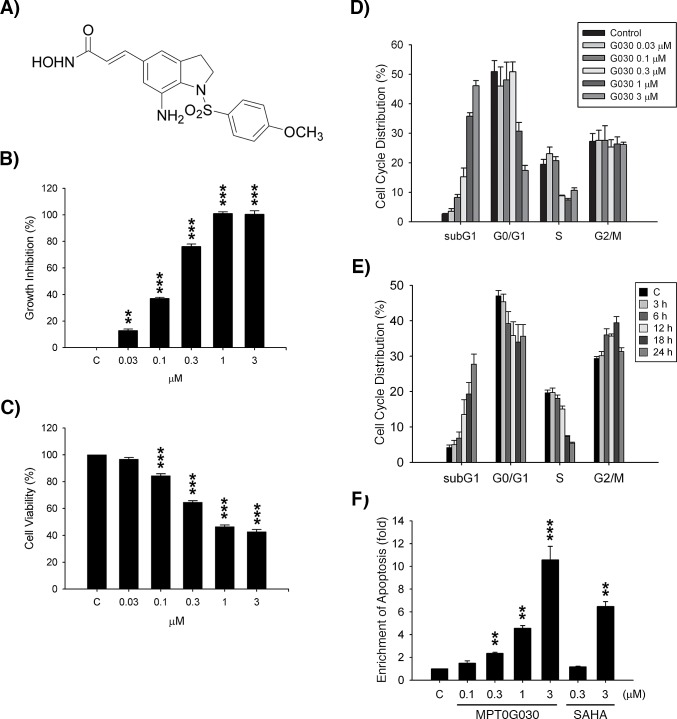
MPT0G030 inhibits growth and induces apoptosis in HT-29 cells (A) The chemical structure of MPT0G030 (3-[7-amino-1-(4-methoxy-benzenesulfonyl)-2,3-dihydro-1H-indol-5-yl]-N-hydroxy-acrylamide). (B) Human colorectal cancer HT-29 cells were incubated with indicated concentrations of MPT0G030 for 48 h, and cell number was determined using SRB assay. (C) Cells were incubated with indicated concentrations of MPT0G030 for 24 h. Then, cell viability was measured by the mitochondrial MTT reduction activity assay. (D, E) HT-29 cells were incubated with MPT0G030 for 24 h for indicated concentrations (D), or MPT0G030 1μM for indicated times (E). Subsequently, cells were analyzed by flow cytometry to determine cell cycle distribution patterns. Quantitative data were based on histograms. (F) Cells were treated with indicated concentrations of MPT0G030 and SAHA for 24 h, and then measured DNA fragmentation with cell death detection ELISA kit. Data are expressed as means ± SEM of three independent experiments.

MPT0G030 (3-[7-amino-1-(4-methoxy-benzenesulfonyl)-2,3-dihydro-1H-indol-5-yl]-N-hydroxy-acrylamide) (Figure 1A) is a novel class I HDAC inhibitor that shows broad-spectrum cytotoxicity against various human cancer cell lines. However, the molecular action mechanism of class I HDAC inhibitor in colorectal cancer has not been clearly elucidated. The studies described here reveal the signaling mechanisms responsible for MPT0G030-induced cell differentiation and apoptosis in human colorectal HT-29 cancer cells.

## RESULTS

### MPT0G030 inhibits cell growth and induces cell death in human colorectal cancer HT-29 cells

To evaluate the biological effects of MPT0G030, human colorectal cancer HT-29 cells were grown in the absence or presence of indicated concentrations of MPT0G030 for 24 or 48 h. As revealed by the sulforhodamine B assay (SRB) assay, MPT0G030 affected cell growth inhibition in a concentration-dependent manner, not only in the HT-29 cells (Figure 1B, GI_50_ = 0.145 ± 0.01 μM), but also in various other cancer cell lines (Supplementary Table 1). MPT0G030's cytotoxicity effect was measured using MTT assay, Figure 1C showed that MPT0G030 caused 50% cell death at a concentration of 1 μM or higher (IC_50_ = 0.985 ± 0.06 μM). To verify whether MPT0G030-induced cell death is concomitant with any alteration of cell cycle distribution, FACScan flow cytometry was performed following MPT0G030 treatment with concentrations and times as indicated. MPT0G030 induced a significant increase in the number of apoptotic cells (subG_1_ phase) in concentration- and time-dependent manners (Figure 1D and 1E). In addition, to distinguish whether MPT0G030-induced cell death is due to apoptosis or necrosis, a photometric enzyme immunoassay was conducted. The data showed that MPT0G030 increased the relative amount of cytoplasmic histone-associated DNA fragments in a concentration-dependent manner (Figure 1F), supporting the hypothesis that MPT0G030 induced apoptosis in HT-29 cells. Compared with SAHA (suberanilohydroxamic acid), which is the HDAC inhibitor currently approved by the U.S. Food and Drug Administration for cancer therapy drug, MPT0G030 showed more effective on inducing cell apoptosis. These results confirmed that MPT0G030 inhibited proliferation and induced apoptosis in HT-29 cells.

### MPT0G030 inhibits histone deacetylase (HDAC) activity

**Figure 2:**
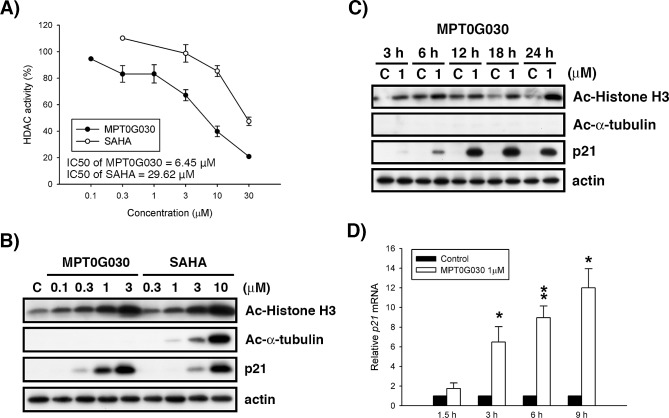
MPT0G030 inhibits histone deacetylase (HDAC) activity (A) HT-29 cells were treated with indicated concentrations of MPT0G030 and SAHA for 24 h; the inhibition effect to total HDACs was assayed by fluorometric HDAC activity assay kit. The HDAC activity (%) was compared with control (calculated as 100 %). (B, C) HT-29 cells were treated with vehicle (0.1% DMSO) or different concentrations of compounds (MPT0G030, SAHA) for indicated times. Then, cells were harvested for detection of acetyl-histone H3, acetyl-α-tubulin and p21 by Western blot analysis. (D) p21 mRNA was analyzed with quantitative real-time RT-PCR after 1.5, 3, 6, 9 h treatment of MPT0G030. Data are expressed as means ± SEM of three independent experiments.

MPT0G030 was designed as a HDAC inhibitor; its ability to inhibit HDACs was evaluated using two different fluorometric HDAC activity assays under varying conditions (with or without cells, total HDACs or individual HDAC isoforms). Relative to SAHA, MPT0G030 showed significantly higher inhibition of total HDACs in HT-29 cells with its IC_50_ being 6.45 ± 1.39 μM (Figure 2A, IC_50_ of SAHA = 29.62 ± 2.62 μM). HDACs are divided into four classes based on their sequence homology. To evaluate the inhibition effects of MPT0G030 on individual HDAC isoforms, assays were performed with recombinant HDAC enzymes. MPT0G030 was an effective inhibitor of HDAC1 and HDAC2, demonstrating a respective IC_50_ of 75.07 ± 4.65 nM, and 241.75 ± 47.65 nM (Table 1). MPT0G030 is a markedly stronger inhibitor of HDAC1 than is SAHA. Interestingly, by contrast to SAHA, MPT0G030 (IC_50_ = 4368.21 ± 42.07 nM) has almost no ability to inhibit HDAC6 (IC_50_ of SAHA= 78.31 ± 8.96 nM) (Table 1).

**Table 1:** The inhibition effect of MPT0G030 to individual HDAC isoforms

Class	HDACs	MPT0G030	Ratio^a^	SAHA	Ratio^a^
Class I	HDAC1	75.07 ± 4.65 nM	1	115.95 ± 5.51 nM	1
	HDAC2	241.75 ± 47.65 nM	3.22	162.87 ± 2.05 nM	1.40
	HDAC8	3344.43 ± 129.99 nM	44.55	5741.27 ± 162.56 nM	49.51
Class IIa	HDAC4	> 10000 nM	>130	> 10000 nM	>130
	HDAC5	> 5000 nM	>60	> 10000 nM	>130
	HDAC7	> 10000 nM	>130	> 10000 nM	>130
	HDAC9	> 10000 nM	>130	> 10000 nM	>130
Class IIb	HDAC6	4368.22 ± 42.07 nM	58.19	78.31 ± 8.96 nM	0.68

Ratio^a^: fold x IC_50_ relative to HDAC1 isoform

Inhibition of Class I HDACs is linked to upregulation of histone H3 acetylation and p21 mRNA and protein expression [2], which have been associated with anti-proliferative activity. It has also been reported that HDAC6 functions as an α-tubulin deacetylase, modulating tubulin stability [2]. We decided to further investigate the relationship between protein expression and function of MPT0G030. As shown in Figure 2B and 2C, accumulation of acetylated histone H3 was accompanied by increased expression of p21 protein level in MPT0G030-treated HT-29 cells concentration- and time-dependently, but no acetyl-α-tubulin was detected. In addiction, p21 mRNA was also upregulated before p21 protein was detectable (Figure 2D). These results suggest that, in contrast to the pan-HDAC inhibitor SAHA, MPT0G030 is an effective and selective class I HDACs inhibitor.

### MPT0G030 alters cell morphology and E-cadherin distribution

**Figure 3:**
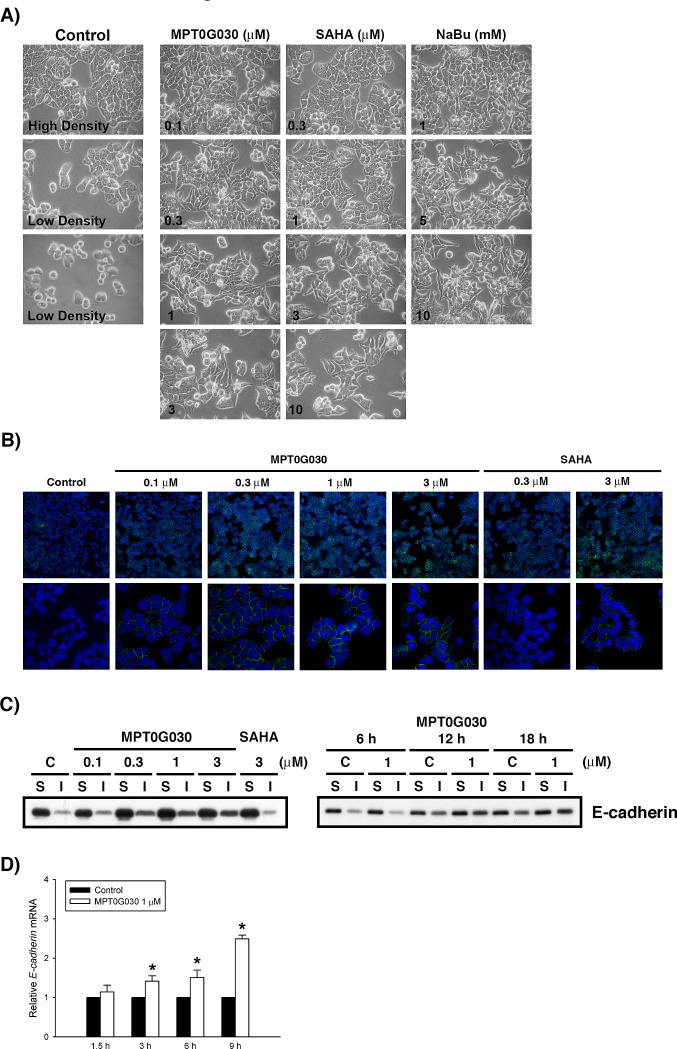
MPT0G030 alters cell morphology and E-cadherin distribution (A) After treatment with MPT0G030 and two different HDAC inhibitors (SAHA and Sodium butyrate (NaBu)) for 18 h, the change of HT-29 cell morphology was observed by phase-contrast light microscopy. (B) After treatment of vehicle (0.1% DMSO) or different concentrations of compounds (MPT0G030 and SAHA) for 18 h, HT-29 cells were stained with E-cadherin and examined by fluorescent microscope. DAPI is in blue and E-cadherin in green. (C) Cells were treated with indicated concentrations and times, then soluble and insoluble (cytoskeletal) fractions of E-cadherin were prepared as described in MATERIALS AND METHODS. (D) E-cadherin mRNA was analyzed with quantitative real-time RT-PCR after 1.5, 3, 6, 9 h treatment of MPT0G030.

The class I HDACs is important regulators of cell proliferation and survival in the colonic proliferating crypt compartment, and they also function as inhibitors of cell maturation. Interestingly, by using phase-contrast light microscopy, we observed that MPT0G030 treatment of colon cancer HT-29 cells changed their morphology in a concentration-dependent fashion. Generally, control HT-29 cells were circular, oval, or slightly polygonal in appearance, even at varying cell densities (Figure 3A). Following incubation with MPT0G030 for 24 h, a decreased cell number and the appearance of floating cells reflected the cytotoxic effect of MPT0G030-induced apoptosis (Figure 3A). Meanwhile, Figure 3A showed that HT-29 cell morphology was altered, changing from circular to columnar and elongated (the so-called dome-like structure), which is a typical feature of the induction of intestinal epithelial cell differentiation [15]. During differentiation, the adherens junction protein E-cadherin is important in maintaining cell structures; functional E-cadherin is associated with the cytoskeleton stability and is located in cell-cell adhesion [16]. By using immunofluorescence analysis, we found E-cadherin was significantly redistributed in response to MPT0G030 treatment (Figure 3B). Translocation of the E-cadherin protein was detected by concentration- and time-dependent increases in cytoskeleton fraction following incubation with different concentrations of MPT0G030 and over time (Figure 3C). Additionally, E-cadherin mRNA was upregulated by treatment with MPT0G030 (Figure 3D). These results suggest that MPT0G030 promotes differentiation and changes the morphology of colon cancer cells through E-cadherin redistribution.

### Activation of PKCδ contributes to MPT0G030-induced cell differentiation and cell death

**Figure 4:**
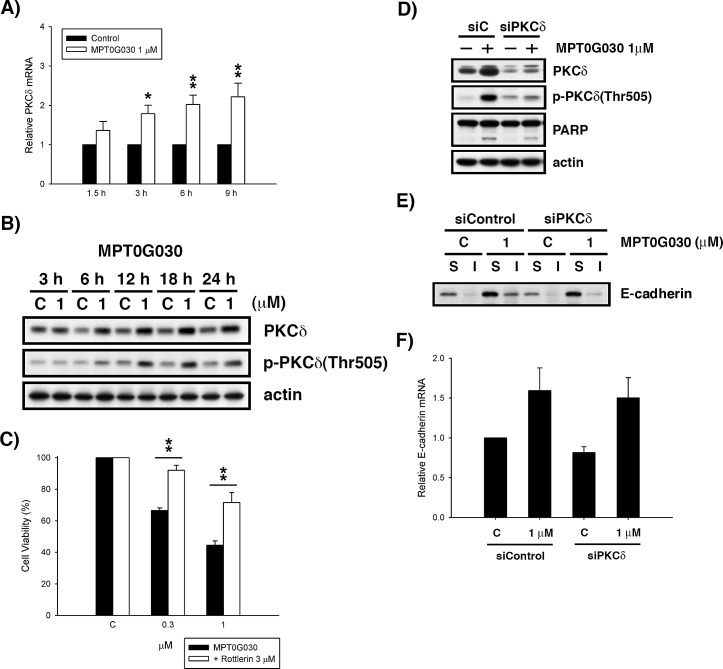
Activation of PKCδ contributes to cell differentiation and cell death (A) PKCδ mRNA was analyzed with quantitative real-time RT-PCR after 1.5, 3, 6, 9 h treatment of MPT0G030. (B) HT-29 cells were treated with vehicle or different concentrations of MPT0G030 for indicated times. Then, cells were harvested for detection of PKCδ and phosphorylated-PKCδ(Thr505) by Western blot analysis. (C) HT-29 cells were incubated with indicated reagents (MPT0G030 and 3 μM Rottlerin) for 24 h. Then, cell viability was determined using MTT assay. (D) Cells were transfected with control siRNA (siC) or PKCδ siRNA (siPKCδ), and then treatment with MPT0G030 for 24 h. And the transfection efficacy and cleavage of PARP were confirmed with Western blot analysis. (E) The E-cadherin distribution was detected in PKCδ siRNA transfected cells after treating with MPT0G030 for 18 h. (F) E-cadherin mRNA was analyzed in PKCδ siRNA transfected cells after 6 h treatment of MPT0G030. Data are expressed as means ± SEM of three independent experiments.

Previous studies have shown PKCδ participates in HDAC inhibitor-induced differentiation of colon cancer and the resultant growth arrest and apoptosis in various cancers, but the mechanisms underlying this effect remains unclear [14, 17]. To investigate whether MPT0G030 activates or regulates PKCδ at the level of transcriptional control in colon cancer cells, HT-29 cells were incubated with MPT0G030 and compared with time-matched controls. MPT0G030 treatment induced PKCδ mRNA expression in a concentration- and time-dependent manner as detected by quantitative PCR (Figure 4A). In addition, PKCδ protein levels also increased with time (Figure 4B). Because the activity of PKCδ is controlled via phosphorylation at conserved serine/threonine sites [10], phosphorylation of PKCδ at threonine 505 was monitored by Western blot. In Figure 4B, PKCδ phosphorylation progressively increased from 6 to 24 hours following MPT0G030 treatment. To further confirm the contribution of PKCδ to MPT0G030-induced biological effects, rottlerin, a specific PKCδ inhibitor, was used to suppress the activation of PKCδ. The combination of MPT0G030 and rottlerin markedly reversed the induction of cell death seen with MPT0G030 alone (Figure 4C). Meanwhile, HT-29 cells were transiently transfected with PKCδ siRNA to deplete its expression. PKCδ siRNA specifically and efficiently downregulated the expression of PKCδ, but control siRNA had no effect. In contrast with control groups, PKCδ siRNA-transfected cells reversed the MPT0G030-induced cleavage of PARP, which as a marker for programmed cell death (cell apoptosis) (Figure 4D). Previous studies have shown that PKCδ plays a critical role in E-cadherin trafficking in epithelial cells [18]. We examined the effect of PKCδ on MPT0G030-induced E-cadherin redistribution. The translocation of E-cadherin was attenuated in PKCδ siRNA-transfected cells (Figure 4E), whereas the E-cadherin mRNA was not affected (Figure 4F). These results suggest that the activation of PKCδ caused by MPT0G030 contributes to both colon cancer differentiation and cell death.

### MPT0G030-induced PKCδ activation is mediated by HDAC1

**Figure 5:**
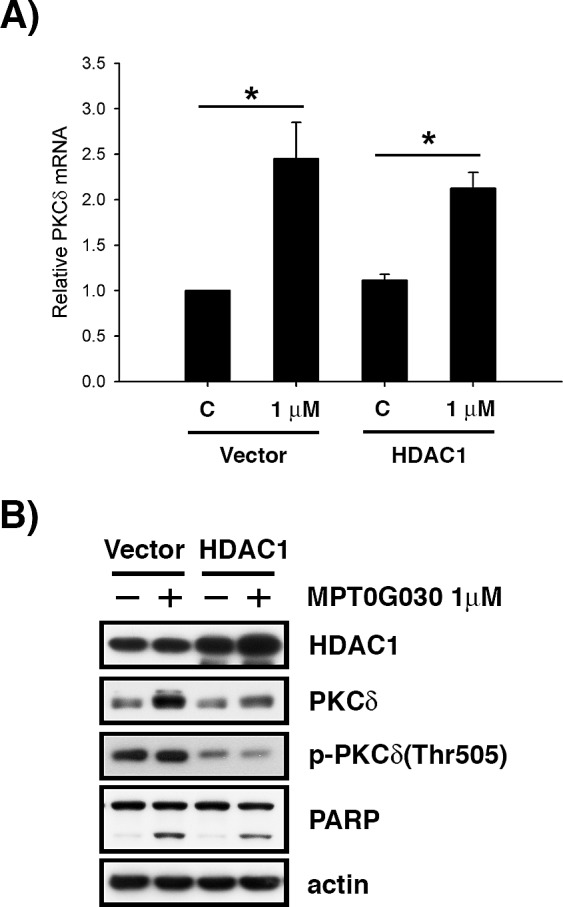
MPT0G030-induced PKCδ activation is mediated by HDAC1 (A) After overexpressing HDAC1 in HT-29 cells, PKCδ mRNA was evaluated by quantitative real-time RT-PCR after 6 h MPT0G030 treatment. (B) Cells were transfected with control vector (vector) or HDAC1 plasmid (HDAC1), and then treatment with MPT0G030 for 24 h. The transfection efficacy and cleavage of PARP, PKCδ and phosphorylated-PKCδ(Thr505) were confirmed with Western blot analysis.

Since MPT0G030 is designed to be an HDAC inhibitor, the contribution of HDACs to MPT0G030-induced PKCδ expression was investigated. This was achieved by evaluating MPT0G030-induced PKCδ mRNA and protein levels after overexpressing HDAC1 in HT-29 cells. We observed that the change of PKCδ mRNA was imperceptible when HDAC1 was overexpressed without MPT0G030 treatment (Figure 5A). Additionally, the increase of PKCδ mRNA was not reversed in HDAC1-overexpressing cells with drug treatment (Figure 5A). However, the protein level and phosphorylation of PKCδ was reduced significantly, and MPT0G030-induced cell apoptosis was also reversed when HDAC1 was overexpressed (Figure 5B). Collectively, these results show that HDAC1 participates in translation and activation of PKCδ, which implicating HDAC1 regulates PKCδ from functional level but not transcriptional level.

### MPT0G030 inhibits HT-29 tumor cell growth in the mouse xenograft models

**Figure 6:**
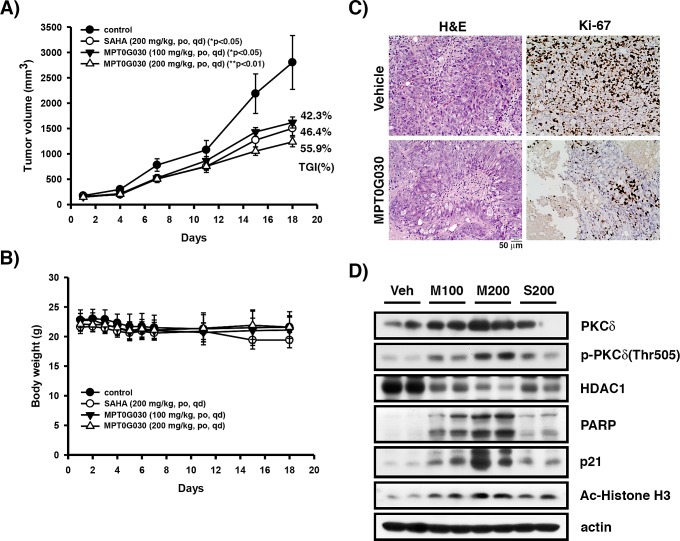
MPT0G030 anticancer activity in HT-29 xenograft models Mice bearing established HT-29 tumors (~100 mm^3^) were divided into four groups (n = 7) and dosed by gavage with vehicle (Veh), MPT0G030 (M100, M200) 100 or 200 mg/kg qd, SAHA (S200) 200 mg/kg qd. (A) The tumor volumes of mice were measured. TGI%: percentages of tumor growth inhibition. (B) The MPT0G030 treatment didn't cause loss of body weight in any groups. (C) Tumor slides that treated with vehicle and MPT0G030 200 mg/kg qd were stained with H&E and Ki-67 protein, then photomicrographs were obtained under × 200 magnification. (D) HT-29 xenograft tumor homogenates were analyzed by Western blots.

To further evaluate the *in vivo* anti-cancer activity of MPT0G030, HT-29 tumor xenograft models were established using athymic nude mice. Mice bearing established HT-29 tumors were treated by oral gavage with vehicle or MPT0G030 (100 mg/kg qd (once every day), 200 mg/kg qd) for the duration of the experiment (18 days), where SAHA (200 mg/kg qd) was used as reference. In contrast to the vehicle-treated group, administration of MPT0G030 resulted in significant inhibition of tumor growth in a dose-dependent manner (Figure 6A). Baseline body weight, which is an indicator of the health of the mice, was not affected by MPT0G030 during the study period, suggesting that mice tolerated the treatment without experiencing evident toxicity *in vivo* (Figure 6B). Furthermore, histological sections of HT-29 xenograft samples were stained with Hematoxylin and Eosin and Ki-67. These experiments revealed that MPT0G030 significantly decreased cell proliferation, of which Ki-67 is a marker (Figure 6C). Tumor homogenates were also prepared for Western blots, and the results showed agreement with the *in vitro* studies (Figure 6D). In particular, HDAC1 was significantly decreased within tumors when treated with MPT0G030 (Figure 6D). Taken together, these findings indicate that MPT0G030 exhibits good ability and advantage as an anti-cancer drug for colon cancer *in vivo* and *in vitro*.

## DISCUSSION

The colonic crypt is the basic functional unit of the human intestine, a highly regenerative organ of the human body formed by a single sheet of colon epithelial cells [19, 20]. Mature differentiated colon cells increase the ability of water absorption and mucus secretion, and then undergo apoptosis at the end of the colonic life cycle [19]. Tumorigenesis in colon epithelial cells is associated with uninhibited tissue regeneration and cell proliferation. The normal physiologic roles of HDACs have been studied for several decades and identified as important tumor promoters that inhibit colon cell maturation and transformation [4]. Abundant literature has shown that treatment with HDAC inhibitors arrests cell growth and stimulates differentiation and apoptosis of colon cancer cells *in vitro* and *in vivo* [4, 6-8, 17]. Therefore, other than existing cytotoxic chemotherapy drugs, HDAC inhibitors may redirect cancer cells back into the normal colonic life cycle of cell differentiation and apoptosis, implicating a rational and promising strategy for colon cancer therapy.

Considerable evidence implies that HDAC inhibitors reprogram cell terminal differentiation and induce apoptosis in colon cancer cells *in vivo* and *in vitro* [4, 8]. Differentiation and apoptosis are physiological processes that are closely linked and in fact inseparable, sharing numerous common features such as chromatin condensation and activation of caspases [21]. Therefore, apoptosis is considered as the endpoint of the differentiated-colonocyte life cycle *in vivo* [17, 19, 22]. In our study, MPT0G030 rapidly induced cell apoptosis after 6-12 h of being administered (Figure 1E), during which redistribution of E-cadherin was detected (Figure 3E). This suggests that apoptosis and differentiation might occur simultaneously in MPT0G030-treated cells.

Previous studies have shown that HDAC inhibitor-induced differentiation is PKCδ-dependent in colon cancer cells [17], and that PKCδ enhances the differentiation and accelerates the apoptosis in PKCδ-overexpressing colon cancer CaCo-2 cells [22]. We observed that PKCδ mRNA and protein levels increased after MPT0G030 treatment (Figure 4A, 4B). The role of PKCδ was further elucidated: our experiment with PKCδ siRNA-transfected cells revealed that E-cadherin distribution was modulated by PKCδ (Figure 4E), but the expression of E-cadherin mRNA was not altered (Figure 4F), implying that PKCδ regulated E-cadherin at the protein functional level. Meanwhile, in the presence of MPT0G030, co-treatment with rottlerin significantly increased cell viability (Figure 4C), and transfection with PKCδ siRNA also reversed PARP cleavage (Figure 4E). These results show that MPT0G030-induced PKCδ participates in cell apoptosis and concomitantly promotes differentiation of colon cancer cells through E-cadherin redistribution and changes in cell morphology.

Taking into account the different epigenetic and genetic expression profiles of colon cancer cell lines, the drug effect of MPT0G030 was also examined in HCT116 cells. HCT116 cell line is known to harbor KRAS mutation, p53 wild type and normal APC; HT-29 has mutated BRAF, p53 and truncated version of APC. Even so, MPT0G030 inhibited HCT116 cell growth effectively (Supplementary Table 1) and increased PKCδ expression and activity (Supplementary Figure 1A). Demonstrating that alteration of PKCδ by MPT0G030 might is general effect for colon cancer but not particular for one cell line.

The E-cadherin-catenin complex plays a critical role in regulating a variety of receptor tyrosine kinases via direct and/or indirect interactions; for instance, as reported for the EGF and Met receptors [23, 24]. In colorectal carcinomas, loss or mislocalization of E-cadherin protein is a functional perturbation rather than a simple protein decrease, resulting in dedifferentiation, invasion and metastasis [25]. The expression pattern of E-cadherin has therefore been identified as an important prognostic factor for assessing colon cancer tumor grade. E-cadherin mRNA was upregulated with MPT0G030 treatment (Figure 3D), and the distribution was evidently at the site of cell-cell adhesion (Figure 3B, 3C). What is more, PKCδ activation plays a critical role in E-cadherin trafficking in epithelial cells via the Met signaling pathway [18, 24]. The tyrosine kinase receptor Met, whose expression is normally confined to epithelium cells, is a proto-oncogene that plays a crucial role in cell regeneration, proliferation, and migration [26]. Abundant evidence demonstrates that c-Met is overexpressed in a large variety of cancer types, especially in cells of epithelial origin [27]. We also investigated Met mRNA and protein expression after MPT0G030 treatment, discovering that the activation of Met was significantly inhibited and Met protein level was progressively decreased (Supplementary Figure 1B). Collectively, MPT0G030 might act by increasing the tumor suppressor PKCδ expression and decreasing the proto-oncogene Met expression via HDAC inhibition, with both these effects contributing to the observed E-cadherin redistribution.

Increasing evidences indicated that histone acetylation by HDAC inhibitors is not the only mechanism of transcriptional activation; post-translational modifications through multiple protein kinases including MAPKs, PI3K, Akt, and PKCδ have also been identified [2, 4, 14]. Our research shows that the activation of PKCδ was inhibited by HDAC1 (Figure 5). In our study, MPT0G030 slightly decreased HDAC1 mRNA but not altered total protein levels of HDAC1 *in vitro* (Data not shown). Therefore, we further focused on the enzyme activity inhibition of HDAC1 caused by MPT0G030 *in vitro*. However, we found that PKCδ was activated and HDAC1 was significantly decreased within tumors after MPT0G030 treatment (Figure 6D), suggesting that MPT0G030 might contribute more to inhibit HDAC1 expression, which was linked to PKCδ activation, in human colorectal HT-29 xenograft. However, many studies indicate that overexpressing individual HDACs do not entirely compensate for the simultaneous inhibition of multiple HDACs by HDAC inhibitor [7]. This may be because transcription-independent effects induced by HDAC inhibitor are equally important in the alteration of gene expression as are in their anticancer activities, linked as they are to biological effects such as apoptosis. Notably, closely related HDACs may compensate for each other to maintain functional integrity; for example, HDAC1 might cover HDAC2 function [28].

However, the unselective pan-HDAC inhibitor SAHA, currently approved for treatment of leukemia and solid tumors, may cause a number of unwanted side effects due to the significance of HDACs in normal physiology [29]. Meanwhile, considering the compensation effect between related HDACs in individual HDACs family, development of drugs that selectively target individual HDACs family has emerged as a new approach in cancer therapy. In this paper, we explored the HDAC inhibition effects of MPT0G030. Compared with SAHA, MPT0G030 is more potent and selective in inhibition of class I HDACs (HDAC1, 2 and 8), and especially in its non-targeting of HDAC6 (IC_50_ of MPT0G030 >55 fold x IC_50_ of SAHA, Table 1). In addition, MPT0G030 also selectively induced cytotoxicity in various cancer cells without causing significant toxicity in normal (human umbilical vein endothelial) cells; the IC_50_ value of MPT0G030 in normal cells was about thirty times higher than that in HT-29 cells (Supplementary Figure 1C; whereas SAHA was about six fold). Recently, the selective HDAC class I inhibitor entinostat (MS-275) has been undergoing phase 1/2 clinical trials targeting various cancers, including Hodgkin's lymphoma, breast cancer and lung cancer. However, according to results seen in HT-29 cells (GI_50_ = 1.29 μM) [30], MPT0G030 may well show better efficacy (GI_50_ = 0.145 ± 0.01 μM). The potential of individual HDACs inhibitors as drug targets in cancer therapy are worthy of consideration. Taken together, our studies indicate that MPT0G030, a class I HDAC inhibitor, has great potential as a new drug candidate for cancer therapy.

## MATERIALS AND METHODS

### Reagents

MPT0G030 (Figure 1A) was designed and synthesized by Professor Jing-Ping Liou's Lab. (School of Pharmacy, College of Pharmacy, Taipei Medical University). RPMI-1640 medium and fetal bovine serum (FBS) were purchased from Invitrogen (Carlsbad, CA, USA). Penicillin-Streptomycin Amphotericin B Solution was obtained from Biological Industries (Beit-Haemek, Israel). Antibodies against various proteins were purchased from following sources: PKCδ and phosphor-PKCδ (Cell Signaling, Beverly, MA, USA); caspase-3 (Imgenex Corporation, San Diego, CA, USA); PARP-1/2 (Santa Cruz Biotechnology, Santa Cruz, CA, USA); actin (Millipore, Bedford, MA, USA); goat anti-rabbit IgG-HRP, goat anti-mouse IgG-HRP (Santa Cruz Biotechnology, Santa Cruz, CA, USA); goat anti-rabbit IgG-FITC (Sigma-Aldrich, St Louis, MO, USA); VECTASHIELD Mounting Medium with DAPI (Vector Laboratories, Burlingame, CA, USA); Propidium iodide, Thiazolyl Blue Tetrazolium Bromide (Sigma-Aldrich, St Louis, MO, USA).

### Cell Culture

The human colorectal cancer cell line HT-29 was obtained from American Type Culture Collection (ATCC). Cells were cultured in RPMI-1640 medium supplemented with 10% heat-inactivated FBS, penicillin 100 U/ml, streptomycin 100 μg/ml, and amphotericin B 2.5 μg/ml. Cells were maintained at 37°C in a humidified incubator with 5% CO_2_/95% air.

### Sulforhodamine B (SRB) Assay

Cells were inoculated into 96-well plates in growth medium. After 24 h, cells were fixed with 10% trichloroacetic acid (TCA), to represent a measurement of the cell population at the time of drug addition (T_z_). After incubation with vehicle (0.1% DMSO) or different concentrations of compound for 48 h (T_t_), cells were fixed with 10% TCA and stained with 0.4% SRB. After staining, unbound SRB was removed by 1% acetic acid. Bound SRB was solubilized with 10 mM trizma base and the absorbance was measured at 515 nm. Percentage of growth inhibition was calculated as: 100−[(T_t_-T_z_)/(C-T_z_)] ×100. T_t_ represents absorbance of compound-treated group, and C means absorbance of vehicle-treated group.

### MTT Assay

Cells were seeded in 96-well plates in growth medium overnight. Cells were treated with vehicle (0.1% DMSO) or different concentrations of compound for 24 h, then incubated with MTT reagent at 37°C for 1 h. After incubation, the insoluble formazan produced in living cells. The formazan crystals were dissolved with DMSO and the absorbance was measured at 550 nm.

### FACScan Flow Cytometric Assay

HT-29 cells were exposed to vehicle (0.1% DMSO) or different concentrations of MPT0G030 for indicated time and then fixed with 70% (v/v) ethanol at −20°C overnight. Cells were washed with PBS and resuspended in PI solution containing propidium iodide (80 μg/ml) and RNase A (100 μg/ml) at room temperature. Then, cellular DNA content was detected with FACScan flow cytometry (BD Biosciences). Cells were counted and cell cycle was analyzed by CellQuest software (BD Biosciences).

### Fluorometric HDAC Activity Assay

Two different types of *in vitro* HDAC activity assays were used (with or without cells). Test compounds were incubated with individual recombinant HDAC isoforms (HDAC 1-9) and the assay was performed according to the manufacturer's protocol (BPS Biosciences, USA). HT-29 cells were treated with MPT0G030 and SAHA for 24 h, and then total cell lysates were analyzed with Fluorometric HDAC Activity Assay Kit (BioVision, USA).

### Western Blotting and Cell Extraction

Cells were treated with vehicle (0.1% DMSO) or different agents for indicated time and harvested by trypsinization. Resuspend the cell pellet in ice-cold cell lysis buffer [20 mM Tris (pH 7.4), 150 mM NaCl, 1 mM EDTA,1 mM EGTA, 1% Triton X-100, 1 mM phenylmethylsulfonyl fluoride, 1 mM Na_3_VO_4_, 5 mM NaF, 10 μg/mL leupetin, 10 μg/mL aprotinin]. The cell lysate was centrifuged after incubation on ice for 15 min. Soluble and insoluble (cytoskeletal) fractions were prepared as previous described [16, 31]. Then, equivalent amounts of protein fractions were loaded and separated by SDS-polyacrylamide gel and transferred onto a nitrocellulose membrane. The membranes were incubated with specific primary antibodies and appropriate secondary antibodies. Detection of signal was performed with chemiluminescence detection kit (Visual Protein Biotechnology, Taipei, Taiwan).

### Transient Transfection

Silencer select siRNA against PKCδ was purchased from Ambion (Austin, TX, USA). HT-29 cells were transfected with Lipofectamine RNAiMAX Transfection Reagent (Invitrogen) according to the manufacturer's protocol. Transfected cells were grown at 37°C then harvested for Western blot analysis.

### Immunofluorescence Analysis

Cells were grown on 8-well chamber slides and treated with vehicle (0.1% DMSO) or different concentrations of compound for 24 h. Cells were fixed with ice-cold methanol and incubated with 2% BSA/PBS blocking buffer. E-cadherin was stained with anti-E-cadherin primary antibody and anti-rabbit IgG-FITC secondary antibody. Subsequently, mounted the slide in VECTASHIELD Mounting Medium and sealed the edges. Photomicrographs were taken using the Carl Zeiss LSM 510 Meta Confocal Microscope.

### Quantitative RT-PCR

Total RNA was extracted with TRIzol reagent by the manufacturer's protocol (Invitrogen, USA). 5 μg mRNA was incubated with random primer at 65°C for 5 min, then mixed with M-MLV RT at 37°C for 1 h to obtain cDNA. The SYBR Green PCR reaction (SYBR® Green PCR Master Mix; Roche, Switzerland) was used to evaluate the amplification of p21, c-Met, PKCδ and E-cadherin genes. Primer sequences used for amplification are as follows: p21, 5'-GGC AGA CCA GCA TGA CAG ATT-3' and 3'-GCG GAT TAG GGC TTC CTC T-5'; PKCδ, 5'-AAC CAT GAG TTT ATC GCC ACC-3' and 3'-AGC GTT ACA TTG CCT GCA TTT-5'; E-cadherin, 5'-ATT CTG ATT CTG CTG CTC TTG-3' and 3'-AGT CCT GGT CCT CTT CTC C-5'. Fluorescent signal was detected and recorded by StepOne Real-Time PCR System (Applied Biosystems, USA), and each amplification reaction was checked for the absence of nonspecific PCR products by melting curve. Relative fold changes in gene expression are calculated as 2^-ΔΔCt^.

### Cell Death Detection ELISA Kit

Cell Death Detection ELISA Kit (Roche Diagnostics, Switzerland) was used to analyze drug-induced apoptosis. HT-29 cells were treated with MPT0G030 for 24h. Adherent cells were collected and the assay was performed according to the manufacturer's protocol.

### *In vivo* HT-29 xenograft studies

HT-29 cells were injected subcutaneously into athymic nude mice to establish HT-29 xenograft model (~100 mm^3^), and then divided into four groups (n = 7) and dosed by gavage with (a) vehicle (1% carboxymethyl cellulose + 0.5% Tween 80), (b) MPT0G030 100 mg/kg qd (once every day), (c) MPT0G030 200 mg/kg qd, (d) SAHA 200 mg/kg qd. Tumor volumes were calculated using caliper measurements twice per week using the formula volume (mm^3^) = (length × width^2^)/2. Body weights were measured daily during the first week and twice per week thereafter. Mice were housed under standard conditions (12 h light/12 h dark at 21~23 °C and 60~85% humidity) with ad libitum access to sterilized food and water. The protocols of the *in vivo* study were approved by the Animal Care and User Committee at National Taiwan University.

### Statistical Analysis

Every result was obtained in at least three independent experiments. Data are expressed as means ± SEM. Statistical analysis was performed with *t*-test and *P*-values <0.05 were considered significant (*P<0.05, **P<0.01, ***P<0.001).

## SUPPLEMENTARY MATERIAL TABLE AND FIGURE


