# Supplementary material for: A novel class I HDAC inhibitor, MPT0G030, induces cell apoptosis and differentiation in human colorectal cancer cells via HDAC1/PKCδ and E-cadherin
Source: Oncotarget. 2014 Jul 1;5(14):5651–62. doi: 10.18632/oncotarget.2155 (PMC4170623; doi:10.18632/oncotarget.2155)
Supplement: Supplementary file 1 [file oncotarget-05-5651-s001.pdf]

A novel class I HDAC inhibitor, MPT0G030, induces cell apoptosis and differentiation in human colorectal cancer cells via HDAC1/PKCδ and E-cadherin

Supplementary Material

Supplementary Table 1: The growth inhibition effect of MPT0G030 to different cancer cell lines.

	HT-29	HCT116	Hep3B	PC3	A549
Compd			GI_{50} (μM ± SE[a])		
MPT0G030	0.16±0.01	0.11 ±0.02	0.14±0.01	0.17±0.01	0.18±0.01
SAHA	0.92±0.05	0.44 ±0.03	0.95±0.04	0.23 ±0.02	2.62±1.37

[a]SE: standard error. All experiments were independently performed at least three times.

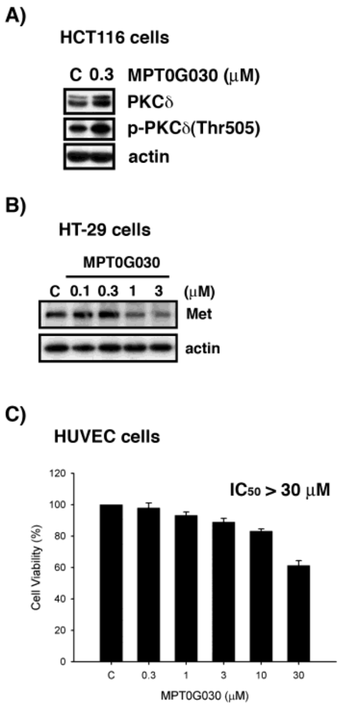

Supplementary Figure 1: (A) HCT116 cells were treated with vehicle or 0.3 μM MPT0G030 for 24h. Then, cells were harvested for detection of PKCδ and phosphorylated-PKCδ(Thr505) by Western blot analysis. (B) HT-29 cells were treated with vehicle or different concentrations of MPT0G030 for indicated times. Then, cells were harvested for detection of Met by Western blot analysis. (C) HUVEC cells were incubated with indicated concentrations of MPT0G030 for 24 h. Then, cell viability was measured by the mitochondrial MTT reduction activity assay.
